# Supplementary material for: PNPLA7 mediates Parkin-mitochondrial recruitment in adipose tissue for mitophagy and inhibits browning
Source: Nat Commun. 2025 Jul 19;16:6651. doi: 10.1038/s41467-025-61904-w (PMC12274619; doi:10.1038/s41467-025-61904-w)
Supplement: Supplementary file 1 — Supplementary Information [file 41467_2025_61904_MOESM1_ESM.pdf]

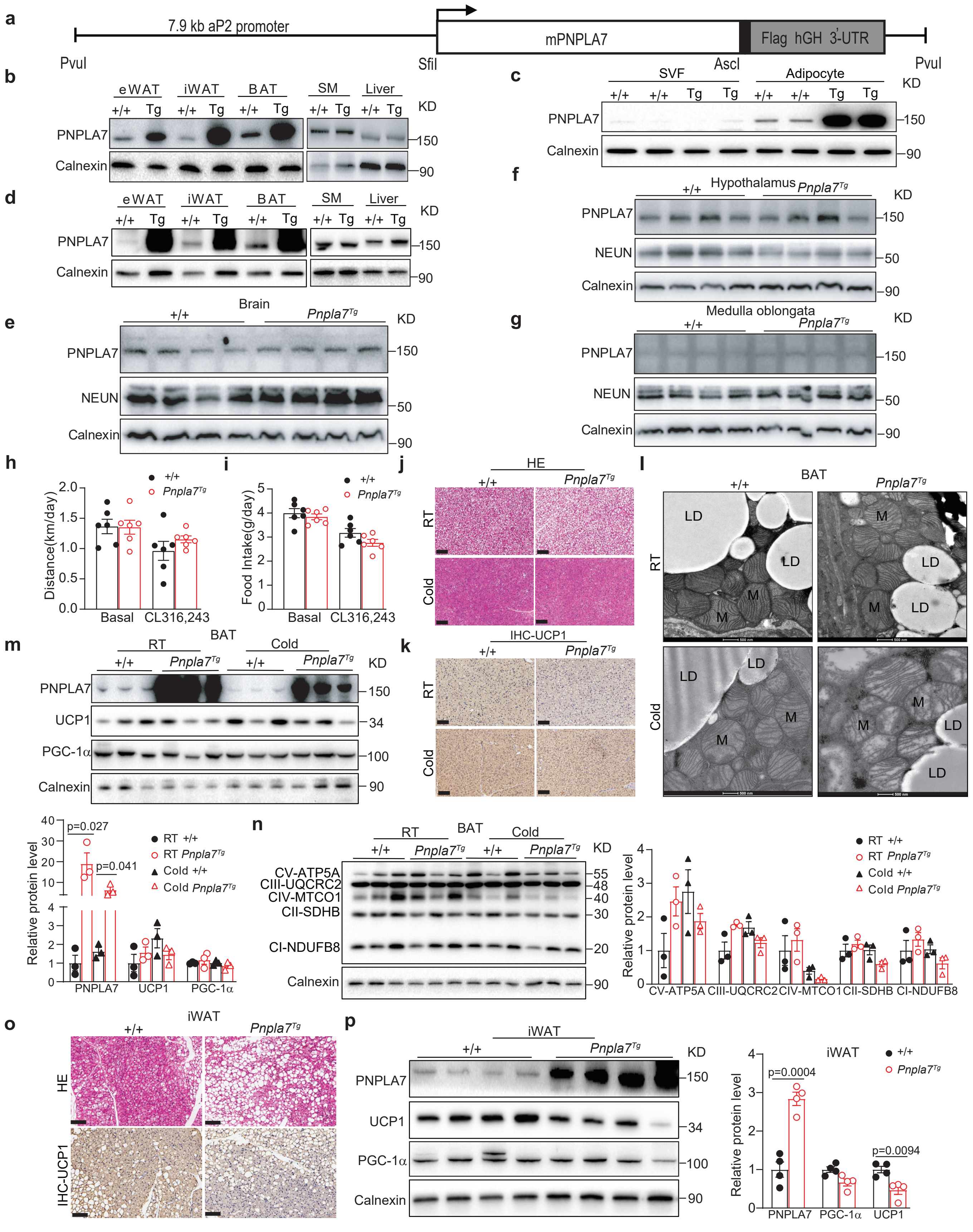

a
7.9 kb aP2 promoter
mPNPLA7
Flag hGH 3'-UTR
PvuI
SfiI
AscI
PvuI
b
eWAT
iWAT
BAT
SM
Liver
PNPLA7
Calnexin
KD
c
SVF
Adipocyte
d
e
Brain
NEUN
f
Hypothalamus
g
Medulla oblongata
h
Distance(km/day)
Basal
CL316,243
i
Food Intake(g/day)
j
HE
k
IHC-UCP1
l
BAT
LD
M
RT
Cold
m
UCP1
PGC-1α
Relative protein level
p=0.027
p=0.041
n
CV-ATP5A
CIII-UQCRC2
CIV-MTCO1
CII-SDHB
CI-NDUFB8
o
iWAT
p
p=0.0004
p=0.0094

Fig.S1. PNPLA7 overexpression has no effect on brown adipose tissue, but inhibits browning of iWAT in the second *Pnpla7*^{Tg} transgenic line during cold exposure

(a) Schematic diagram of the mouse PNPLA7 transgenic construct with Flag-tag. Construct is under the control of *aP2* enhancer/promoter. UTR, untranslated region; mPNPLA7, mouse PNPLA7; Flag, Flag-tag; hGH, human GH. **(b)** Representative Immunoblot results of PNPAL7 protein level in eWAT, iWAT, BAT, Skeletal Muscle (SM) and Liver of 10-week-old control (+/+) and *Pnpla7*^{Tg} (Tg) mice (n=3 biological replicates). **(c)** Representative Immunoblot results of PNPAL7 protein level in stromal vascular fraction (SVF) and differentiated primary SVF-derived adipocytes from iWAT of control (+/+) and *Pnpla7*^{Tg} (Tg) mice. **(d)** Representative Immunoblot results of PNPLA7 protein levels in eWAT, iWAT, BAT, Skeletal Muscle (SM) and Liver of 10-week-old control (+/+) and the second *Pnpla7*^{Tg} transgenic line (n=3 biological replicates). **(e-f)** Representative Immunoblot results of PNPAL7 protein level expression in the brain (**e**), hypothalamus (**f**) and medulla oblongata (**g**) of 10-week-old wild-type control (+/+) and *Pnpla7*^{Tg} (Tg) mice (n=4/group). **(h, i)** Locomotor activity (**h**) and food intake (**i**) analysis of 8-week-old male control and *Pnpla7*^{Tg} mice (n=6/group). Data are presented as mean ± SEM. (Two-tailed Student's *t*-test for 2-group comparisons). **(j, k)** Representative H&E (**j**) and UCP1 immunohistochemical (**k**) staining

images of BAT sections from 10-week-old control (+/+) and *Pnpla7*^{Tg} mice (n=3/group). Scale bar=100 μm. **(l)** Representative TEM images showing mitochondria of BAT from 10-week-old control and *Pnpla7*^{Tg} mice (n=3 biological replicates). Scale bar=500 nm. **(m, n)** Representative Immunoblot results and densitometry analysis of the indicated proteins in BAT of 10-week-old control (+/+) and *Pnpla7*^{Tg} mice (n=3/group). Data are presented as mean ± SEM. (Two-tailed Student's *t*-test for 2-group comparisons). **(o)** Representative H&E and UCP1 immunohistochemical staining images of iWAT sections from 10-week-old control (+/+) and the second *Pnpla7*^{Tg} transgenic line (n=3/group). Scale bar=100 μm. **(p)** Representative Immunoblot results and densitometry analysis of the indicated proteins in iWAT of 10-week-old control (+/+) and the second *Pnpla7*^{Tg} transgenic line (n=4/group). Data are presented as mean ± SEM. (Two-tailed Student's *t*-test for 2-group comparisons).

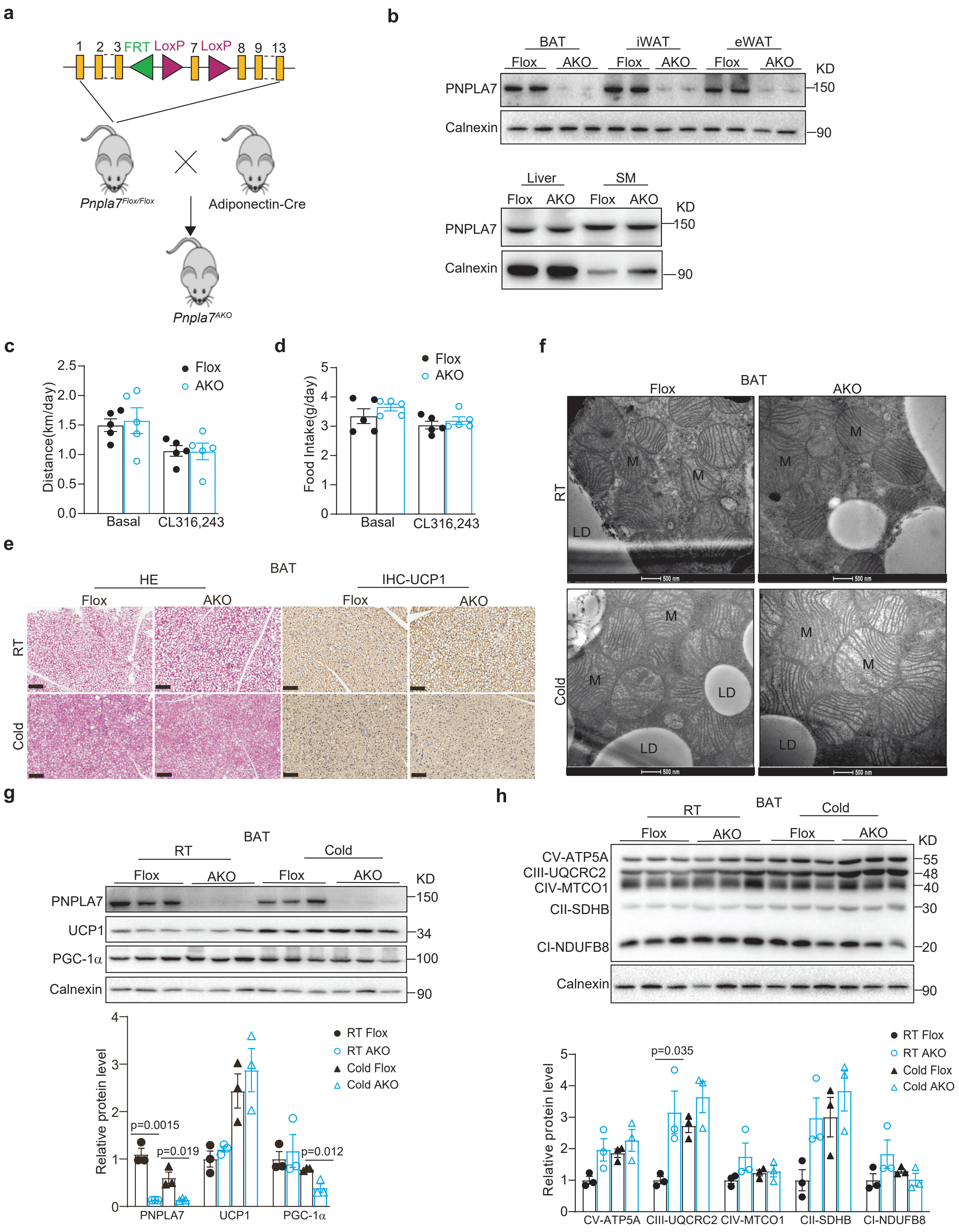
a
1 2 3 FRT LoxP 7 LoxP 8 9 13
Pnpla7Flox/Flox
Adiponectin-Cre
Pnpla7AKO
b
BAT iWAT eWAT
Flox AKO Flox AKO Flox AKO
PNPLA7
Calnexin
KD
150
90
Liver SM
Flox AKO Flox AKO
c
Distance(km/day)
Flox
AKO
Basal CL316,243
d
Food Intake(g/day)
Flox
AKO
Basal CL316,243
e
BAT
HE IHC-UCP1
Flox AKO Flox AKO
RT
Cold
f
BAT
Flox AKO
RT
Cold
M
LD
g
BAT
RT Cold
Flox AKO Flox AKO
PNPLA7
UCP1
PGC-1α
Calnexin
KD
150
34
100
90
Relative protein level
p=0.0015
p=0.019
p=0.012
RT Flox
RT AKO
Cold Flox
Cold AKO
PNPLA7 UCP1 PGC-1α
h
BAT
RT Cold
Flox AKO Flox AKO
CV-ATP5A
CIII-UQCRC2
CIV-MTCO1
CII-SDHB
CI-NDUFB8
Calnexin
KD
55
48
40
30
20
90
Relative protein level
p=0.035
RT Flox
RT AKO
Cold Flox
Cold AKO
CV-ATP5A CIII-UQCRC2 CIV-MTCO1 CII-SDHB CI-NDUFB8

Fig.S2. Adipose tissue specific knockout of *Pnpla7* has minimum effect on the activation of brown adipose tissue during cold exposure.

(a) Schematic diagram of the strategy used to generate *Pnpla7* adipose tissue specific knockout (*Pnpla7*^{AKO}) mice. This illustration was created with Adobe Illustrator, the mouse element from https://pixy.org. **(b)** Representative Immunoblot results of PNPLA7 protein level in different tissues of 10-week-old control (Flox) and PNPLA7 adipose tissue specific knockout (*Pnpla7*^{AKO}) mice under normal chow diet (n=3/group). **(c ,d)** Locomotor activity (**c**) and food intake(**d**) analysis of 8-week-old male control and *Pnpla7*^{AKO} mice (n=5/group). Data are presented as mean ± SEM. (Two-tailed Student's *t*-test for 2-group comparisons). **(e)** Representative H&E and UCP1 immunohistochemical staining images of BAT sections from control (Flox) and *Pnpla7*^{AKO} mice (n=3/group). Scale bar=100 μm. **(f)** Representative TEM images showing mitochondria of BAT from control (Flox) and *Pnpla7*^{AKO} mice (n=3 biological replicates). Scale bar=500 nm. **(g)** Representative Immunoblot results and densitometry analysis of the indicated proteins in BAT of control (Flox) and *Pnpla7*^{AKO} mice (n=3/group). Data are presented as mean ± SEM. (Two-tailed Student's *t*-test for 2-group comparisons). **(h)** Representative Immunoblot results and densitometry analysis of mitochondrial OXPHOS protein levels in BAT of 10-week-old male Flox and *Pnpla7*^{AKO} male mice. Representative results from 3 independent experiments were shown. Data

are presented as mean ± SEM. (Two-tailed Student's *t*-test for 2-group comparisons).

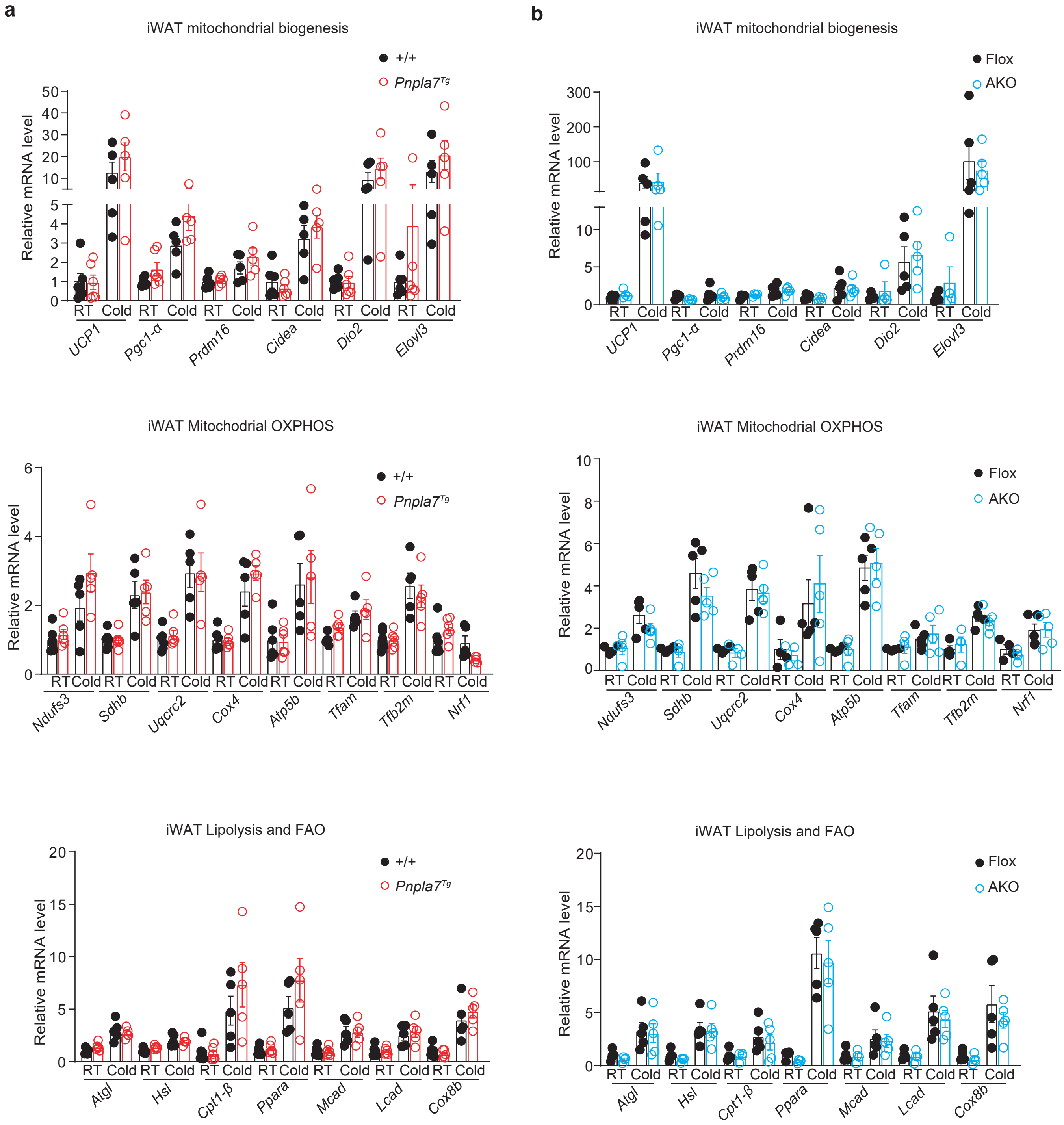
a
iWAT mitochondrial biogenesis
+/+
Pnpla7Tg
Relative mRNA level
RT Cold
UCP1
Pgc1-α
Prdm16
Cidea
Dio2
Elovl3
iWAT Mitochondrial OXPHOS
Ndufs3
Sdhb
Uqcrc2
Cox4
Atp5b
Tfam
Tfb2m
Nrf1
iWAT Lipolysis and FAO
Atgl
Hsl
Cpt1-β
Ppara
Mcad
Lcad
Cox8b
b
iWAT mitochondrial biogenesis
Flox
AKO
iWAT Mitochondrial OXPHOS
iWAT Lipolysis and FAO

Fig.S3. Manipulation of PNPLA7 expression levels does not affect the gene expression in iWAT after prolonged cold exposure.

(a) Quantitative PCR of indicated mRNA levels of genes involved in mitochondrial biogenesis, mitochondrial OXPHOS, and lipolysis or fatty acid β-oxidation in iWAT of control (+/+) and $Pnpla7^{Tg}$ mice after prolonged cold exposure at 6 °C for 7 days under normal chow diet (RT: n =6/group; cold: n=5/group). Data are presented as mean ± SEM. (two-tailed Student's t-test for 2-group comparisons). **(b)** Quantitative PCR of indicated mRNA levels of genes involved in mitochondrial biogenesis, mitochondrial OXPHOS, and lipolysis or fatty acid β-oxidation in iWAT of male Flox and $Pnpla7^{AKO}$ mice after prolonged cold exposure at 6 °C for 7 days under normal chow diet (RT: n=4/group; cold: n=5/group). Data are presented as mean ± SEM. (two-tailed Student's t-test for 2-group comparisons).

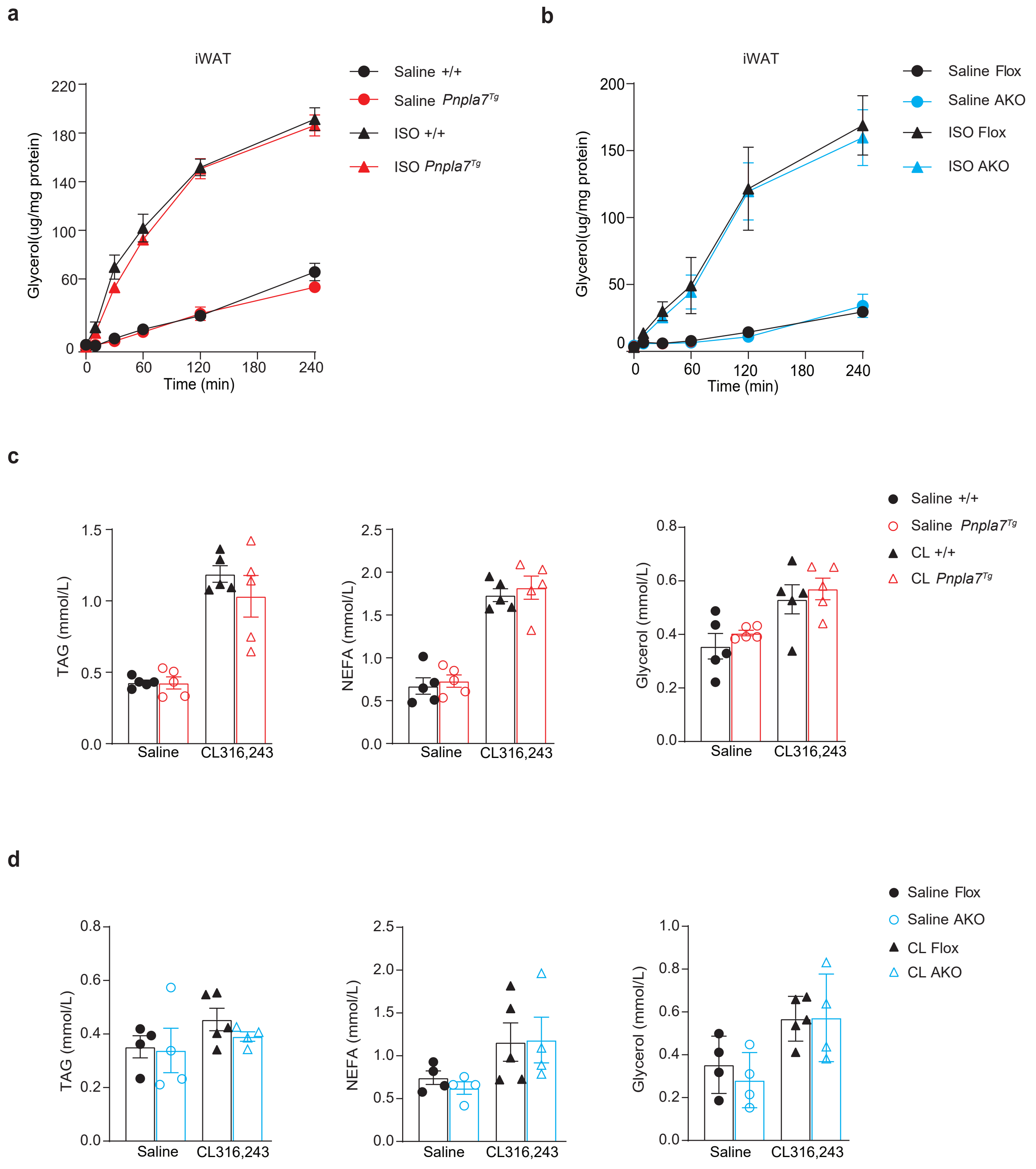

a
iWAT
Glycerol(ug/mg protein)
Time (min)
Saline +/+
Saline Pnpla7Tg
ISO +/+
ISO Pnpla7Tg
b
iWAT
Glycerol(ug/mg protein)
Time (min)
Saline Flox
Saline AKO
ISO Flox
ISO AKO
c
TAG (mmol/L)
NEFA (mmol/L)
Glycerol (mmol/L)
Saline
CL316,243
Saline +/+
Saline Pnpla7Tg
CL +/+
CL Pnpla7Tg
d
TAG (mmol/L)
NEFA (mmol/L)
Glycerol (mmol/L)
Saline
CL316,243
Saline Flox
Saline AKO
CL Flox
CL AKO

Fig.S4. PNPLA7 has no effect on lipolysis ex vivo and in vivo.

(a) *Ex vivo* lipolysis assay performed on isolated iWAT of 10-week-old control (+/+) and *Pnpla7*^{Tg} mice following saline or isoproterenol (10 μM) treatment for the indicated time (Saline: +/+ group: n=4; *Pnpla7*^{Tg} group: n=3; CL: n=4/group). Data are presented as mean ± SEM. (two-tailed Student's t-test for 2-group comparisons). **(b)** *Ex vivo* lipolysis assay performed on isolated iWAT of 10-week-old control (Flox) and *Pnpla7*^{AKO} mice following saline or isoproterenol (10 μM) treatment for the indicated time (Saline: n=4/group; CL: flox group n=3; *Pnpla7*^{AKO} group: n=4). Data are presented as mean ± SEM. (two-tailed Student's t-test for 2-group comparisons). **(c, d)** Plasma TAG, NEFA and glycerol level in 10-week-old control (+/+) and *Pnpla7*^{Tg} (**c,** n=5/group) as well as control (Flox) and *Pnpla7*^{AKO} (**d,** saline: n=4/group; CL: flox group n=4; *Pnpla7*^{AKO} group: n=5) mice under basal condition (saline) or CL 316,243 stimulation *in vivo*. 4 h post-fasting, mice were injected intraperitoneally with saline or CL 316,243 (1 mg/kg). Blood was collected 6 h after injection for the determination of TAG, NEFA and glycerol levels. Data are presented as mean ± SEM. (two-tailed Student's t-test for 2-group comparisons).

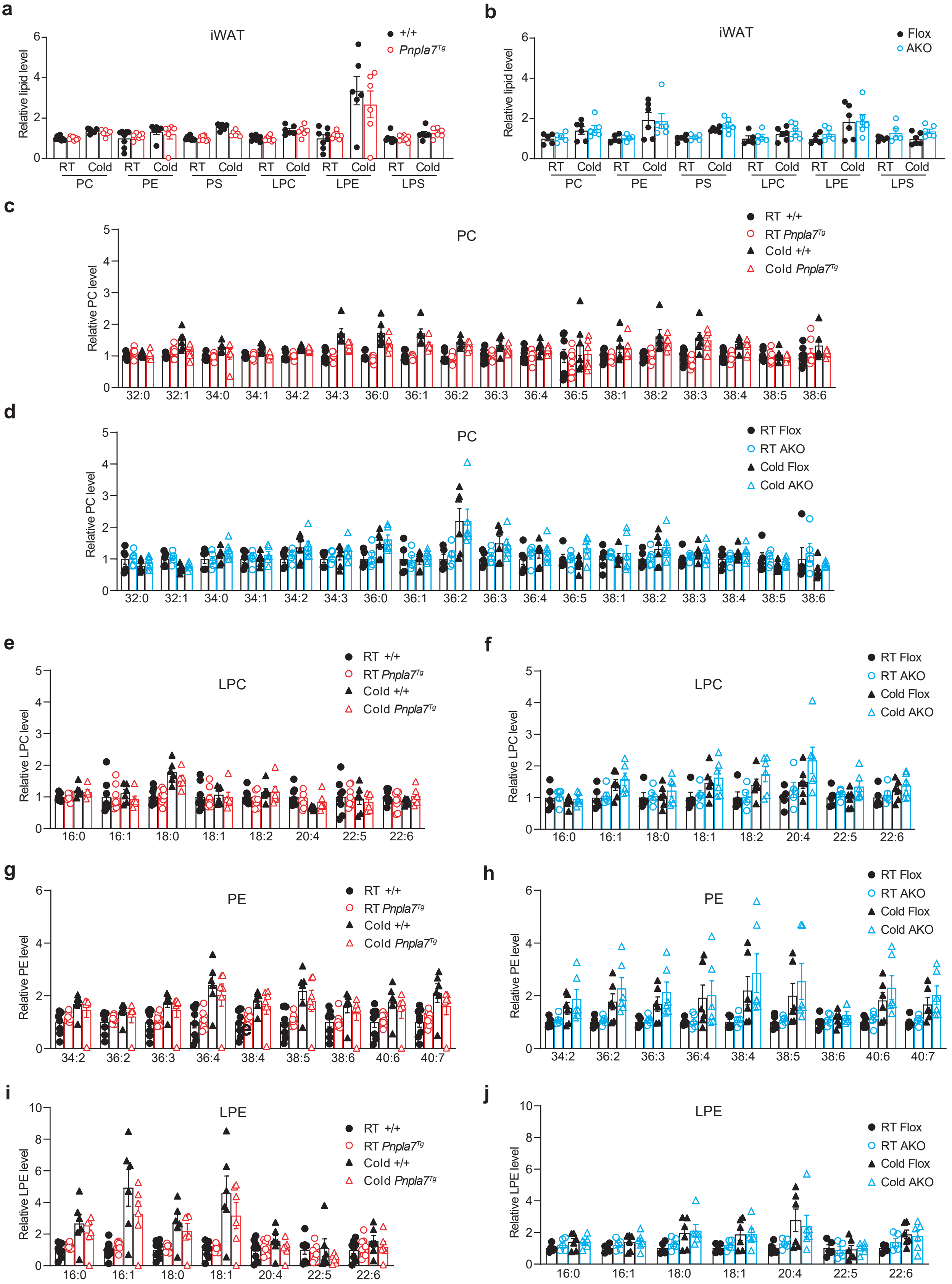
a
iWAT
+/+
Pnpla7^Tg
Relative lipid level
RT Cold RT Cold RT Cold RT Cold RT Cold RT Cold
PC PE PS LPC LPE LPS
b
iWAT
Flox
AKO
Relative lipid level
PC PE PS LPC LPE LPS
c
PC
RT +/+
RT Pnpla7^Tg
Cold +/+
Cold Pnpla7^Tg
Relative PC level
32:0 32:1 34:0 34:1 34:2 34:3 36:0 36:1 36:2 36:3 36:4 36:5 38:1 38:2 38:3 38:4 38:5 38:6
d
PC
RT Flox
RT AKO
Cold Flox
Cold AKO
Relative PC level
e
LPC
Relative LPC level
16:0 16:1 18:0 18:1 18:2 20:4 22:5 22:6
f
LPC
Relative LPC level
g
PE
Relative PE level
34:2 36:2 36:3 36:4 38:4 38:5 38:6 40:6 40:7
h
PE
Relative PE level
i
LPE
Relative LPE level
16:0 16:1 18:0 18:1 20:4 22:5 22:6
j
LPE
Relative LPE level

Fig.S5. PNPLA7 has insignificant effect on lysophospholipid level in iWAT after prolonged cold exposure.

(a, b) PC, PE, PS, LPC, LPE, LPS content in iWAT obtained from control and *Pnpla7*^{Tg} (**a**) or Flox and *Pnpla7*^{AKO} (**b**) male mice under room temperature or prolonged cold exposure at 6 °C for 7 days under normal chow diet (a:RT: n=8/group; cold: n=6/group, b: RT: n=5/group; cold: n=6/group). Data are presented as mean ± SEM. (two-tailed Student's t-test for 2-group comparisons). **(c-f)** Representative content of PC (**c**, **d**) and LPC (**e**, **f**) subclasses in iWAT obtained from control and *Pnpla7*^{Tg} (c, e) or Flox and *Pnpla7*^{AKO} (d, f) male mice under room temperature or prolonged cold exposure at 6 °C for 7 days under normal chow diet (c, e: RT: n=8/group; cold: n=6/group, d, f: RT: n=5/group; cold: n=6/group). Data are presented as mean ± SEM. (two-tailed Student's t-test for 2-group comparisons). **(g-j)** Representative content of PE (**g**, **h**) and LPE (**i**, **j**) subclasses in iWAT obtained from control and *Pnpla7*^{Tg} (g, i) or Flox and *Pnpla7*^{AKO} (h, j) male mice under room temperature or prolonged cold exposure at 6 °C for 7 days under normal chow diet (g, i: RT: n=8/group; cold: n=6/group, h, j: RT: n=5/group; cold: n=6/group). Data are presented as mean ± SEM. (two-tailed Student's t-test for 2-group comparisons).

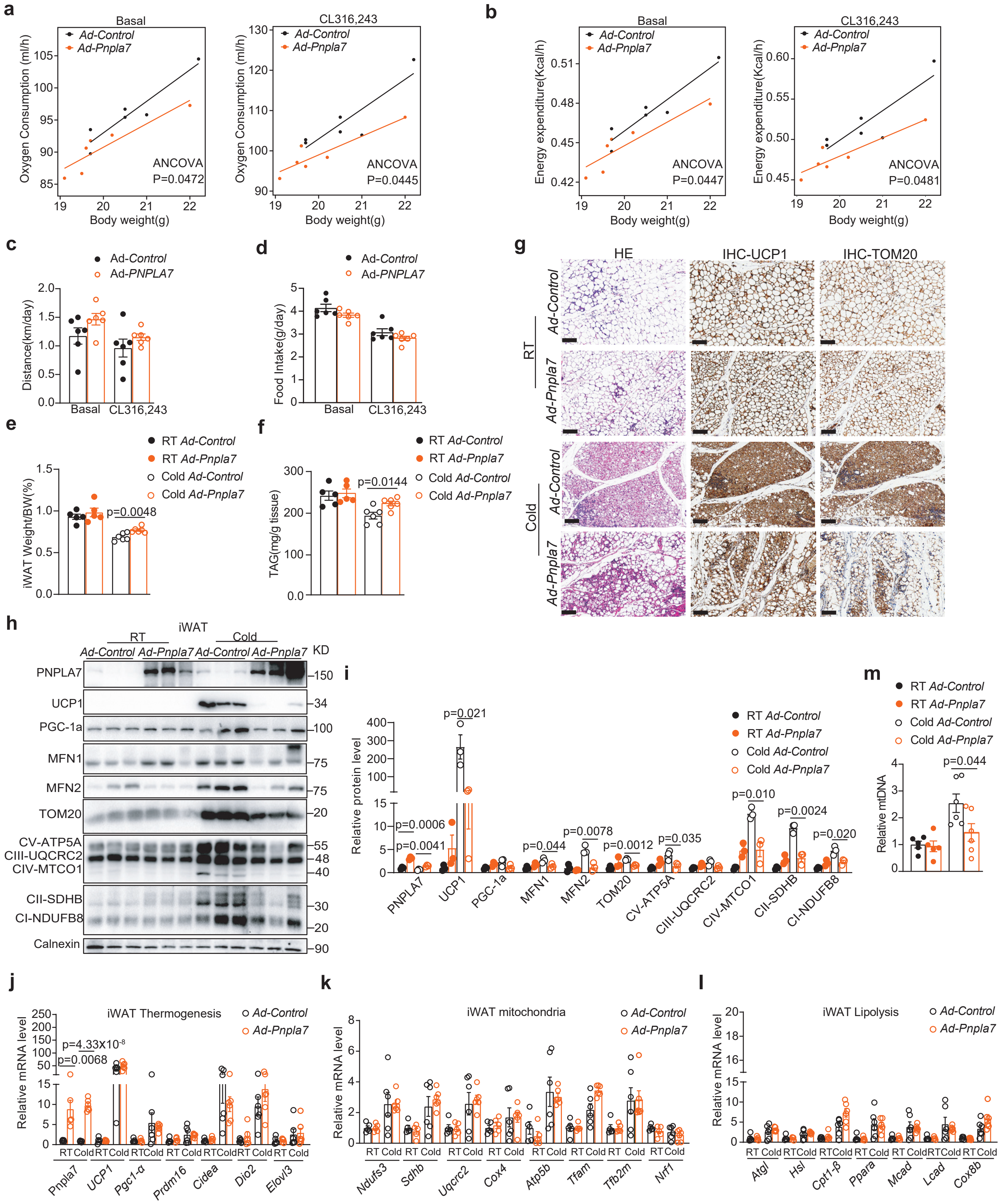

a
Basal
CL316,243
Ad-Control
Ad-Pnpla7
Oxygen Consumption (ml/h)
Body weight(g)
ANCOVA
P=0.0472
P=0.0445
b
Energy expenditure(Kcal/h)
P=0.0447
P=0.0481
c
Ad-Control
Ad-PNPLA7
Distance(km/day)
d
Food Intake(g/day)
e
RT Ad-Control
RT Ad-Pnpla7
Cold Ad-Control
Cold Ad-Pnpla7
iWAT Weight/BW(%)
p=0.0048
f
TAG(mg/g tissue)
p=0.0144
g
HE
IHC-UCP1
IHC-TOM20
RT
Cold
h
iWAT
PNPLA7
UCP1
PGC-1a
MFN1
MFN2
TOM20
CV-ATP5A
CIII-UQCRC2
CIV-MTCO1
CII-SDHB
CI-NDUFB8
Calnexin
KD
i
Relative protein level
p=0.021
p=0.0006
p=0.0041
p=0.044
p=0.0078
p=0.0012
p=0.035
p=0.010
p=0.0024
p=0.020
m
Relative mtDNA
p=0.044
j
iWAT Thermogenesis
Relative mRNA level
p=4.33X10⁻⁸
p=0.0068
Pnpla7
UCP1
Pgc1-α
Prdm16
Cidea
Dio2
Elovl3
k
iWAT mitochondria
Ndufs3
Sdhb
Uqcrc2
Cox4
Atp5b
Tfam
Tfb2m
Nrf1
l
iWAT Lipolysis
Atgl
Hsl
Cpt1-β
Ppara
Mcad
Lcad
Cox8b

Fig.S6. PNPLA7 overexpression in iWAT of wild-type mice impairs browning capacity

8-week-old male *C57BL/6J* mice were orthotopically injected with recombinant adenovirus of Ad-Adiponectin-*mCherry* or Ad-Adiponectin-*Pnpla7* into the subcutaneous inguinal white adipose tissue and fed with normal chow diet for 4 days and subsequently transferred to 6 °C for 7 days to facilitate further investigation. **(a, b)** Regression-based analysis of absolute oxygen consumption (**a**) and energy expenditure (**b**) against body mass from Ad-Adiponectin-*mCherry* (*Ad-control*) and Ad-Adiponectin-*Pnpla7* (*Ad-Pnpla7*) male mice. (n=6/group). Oxygen consumption and energy expenditure as dependent variable, genotype as fixed variable and body mass as covariate. (Two-sided analysis of covariance). **(c, d)** Locomotor activity (**c**) and food intake(**d**) analysis of *Ad-control* and *Ad-Pnpla7* mice (n=6/group). Data are presented as mean ± SEM. (Two-tailed Student's t test for 2-group comparisons). **(e, f)** Weight ratio of iWAT (**e**) and TAG levels of iWAT (**f**) harvested from *Ad-control* and *Ad-Pnpla7* mice. (RT: n=5/group; Cold: n=6/group). Data are presented as mean ± SEM. (Two-tailed Student's t test for 2-group comparisons). **(g)** Representative H&E, UCP1 and TOM20 immunohistochemical staining images of iWAT sections from *Ad-control* and *Ad-Pnpla7* mice (n=3/group). Scale bar=100 μm. **(h, i)** Representative Immunoblot results (**h**) and densitometry analysis (**i**) of the indicated proteins in iWAT

harvested from *Ad-control* and *Ad-Pnpla7* mice (n=3/group). Data are presented as mean ± SEM. (Two-tailed Student's t test for 2-group comparisons). **(j-l)** Quantitative PCR of indicated mRNA levels of genes involved in mitochondrial biogenesis or thermogenesis (**j**), mitochondrial OXPHOS (**k**), and lipolysis or fatty acid β-oxidation (**l**) in iWAT of *Ad-control* and *Ad-Pnpla7* mice (RT: n = 5/group; cold: n=6/group). Data are presented as mean ± SEM. (Two-tailed Student's t test for 2-group comparisons). **(m)** Relative mtDNA content of iWAT harvested from *Ad-control* and *Ad-Pnpla7* mice (RT: n = 5/group; Cold: n=6). Data are presented as mean ± SEM. (Two-tailed Student's t test for 2-group comparisons).

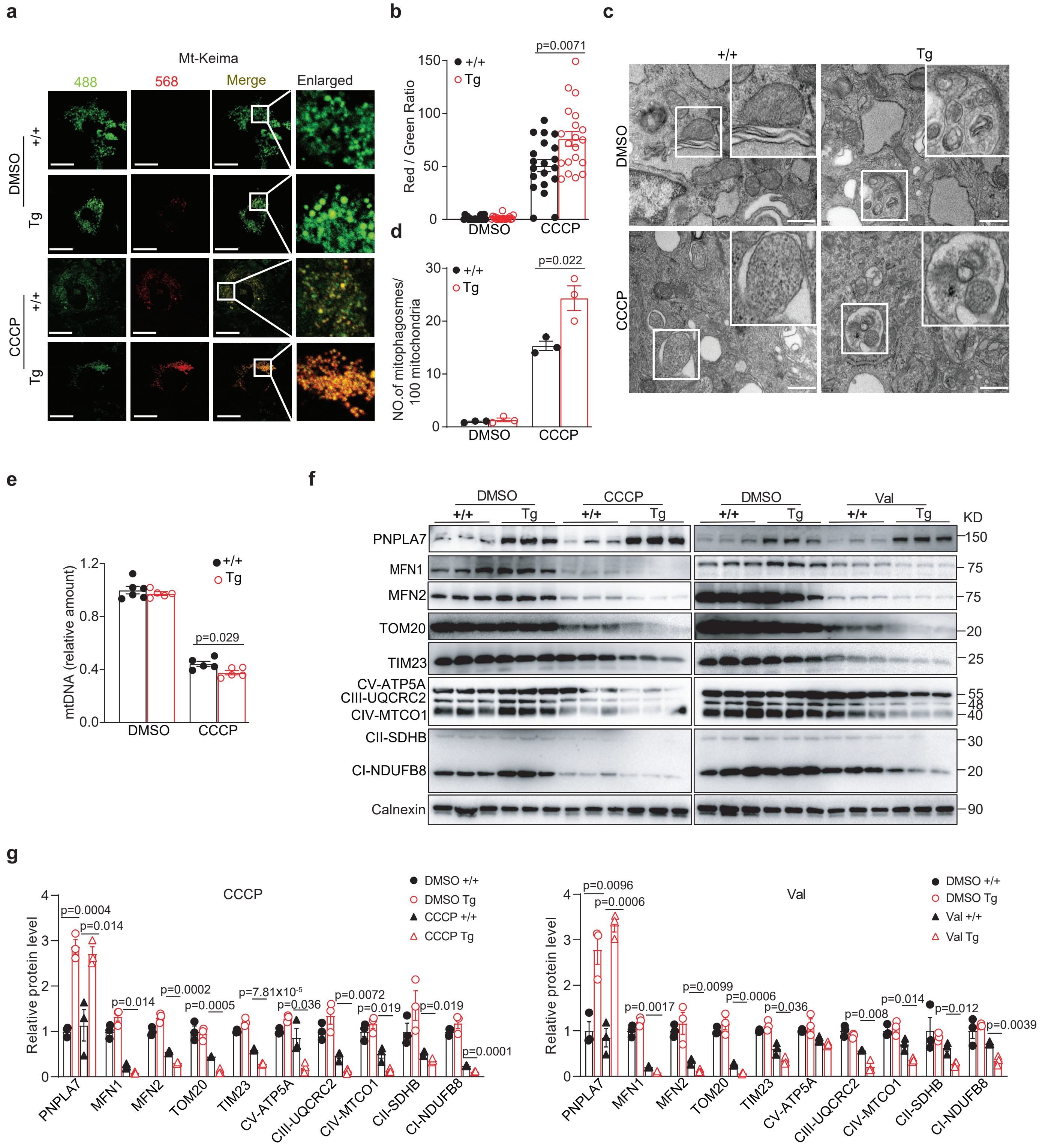
a
Mt-Keima
488
568
Merge
Enlarged
DMSO
+/+
Tg
CCCP
+/+
Tg
b
Red / Green Ratio
+/+
Tg
p=0.0071
DMSO
CCCP
c
+/+
Tg
DMSO
CCCP
d
NO.of mitophagosmes/ 100 mitochondria
+/+
Tg
p=0.022
DMSO
CCCP
e
mtDNA (relative amount)
+/+
Tg
p=0.029
DMSO
CCCP
f
DMSO
CCCP
DMSO
Val
+/+
Tg
KD
PNPLA7
MFN1
MFN2
TOM20
TIM23
CV-ATP5A
CIII-UQCRC2
CIV-MTCO1
CII-SDHB
CI-NDUFB8
Calnexin
150
75
75
20
25
55
48
40
30
20
90
g
CCCP
Relative protein level
DMSO +/+
DMSO Tg
CCCP +/+
CCCP Tg
p=0.0004
p=0.014
p=0.014
p=0.0002
p=0.0005
p=7.81X10-5
p=0.036
p=0.0072
p=0.019
p=0.019
p=0.0001
PNPLA7
MFN1
MFN2
TOM20
TIM23
CV-ATP5A
CIII-UQCRC2
CIV-MTCO1
CII-SDHB
CI-NDUFB8
Val
DMSO +/+
DMSO Tg
Val +/+
Val Tg
p=0.0096
p=0.0006
p=0.0017
p=0.0099
p=0.0006
p=0.036
p=0.008
p=0.014
p=0.012
p=0.0039

Fig.S7. PNPLA7 overexpression promotes mitophagy

Stromal vascular fraction (SVF) isolated from iWAT of 3-week-old control (+/+) and *Pnpla7*^{Tg} (Tg) male mice were differentiated into mature adipocytes *ex vitro*. **(a)** Representative fluorescence image of mitophagy in differentiated adipocytes. Differentiated adipocytes were infected with mito-Keima lentivirus and treated with DMSO or CCCP (10 μM) for 12 h (n=3 biological replicates). The fluorescence images were obtained by confocal microscopy after excited at 488-nm and 568-nm are shown in green and red in the same cell, respectively. Scale bar=20 μm. **(b)** Quantification of the relative ratio of fluorescence intensities (568 nm:488 nm) of the cells described in (**a**, n=20/group). Data are presented as mean ± SEM. (Two-tailed Student's t test for 2-group comparisons). **(c)** Representative TEM images showing mitophagy in differentiated mature adipocytes treated with DMSO or CCCP (10 μM) for 12 h (n=3 biological replicates). White box highlights the mitochondria and mitophagosome. Scale bar=500 nm. **(d)** Quantification of mitophagosome number per 100 mitochondria in the cells described in (**c**). (n=3 independent experiments). Data are presented as mean ± SEM. (Two-tailed Student's t test for 2-group comparisons). **(e)** Relative mtDNA content in differentiated mature adipocytes treated with DMSO or CCCP (10 μM) for 24 h (DMSO: +/+ group: n=6; Tg group: n=5; CCCP: n=5/group). Data are presented as mean ± SEM. (Two-tailed Student's t test for 2-group comparisons). **(f, g)**

Representative Immunoblot results(**f**) and densitometry analysis(**g**) of mitochondrial membrane, and OXPHOS protein levels in differentiated mature adipocytes treated with CCCP (10 μM) or Valinomycin (1 μM) for 24 h. (n=3 biological replicates). Data are presented as mean ± SEM. (Two-tailed Student's t test for 2-group comparisons).

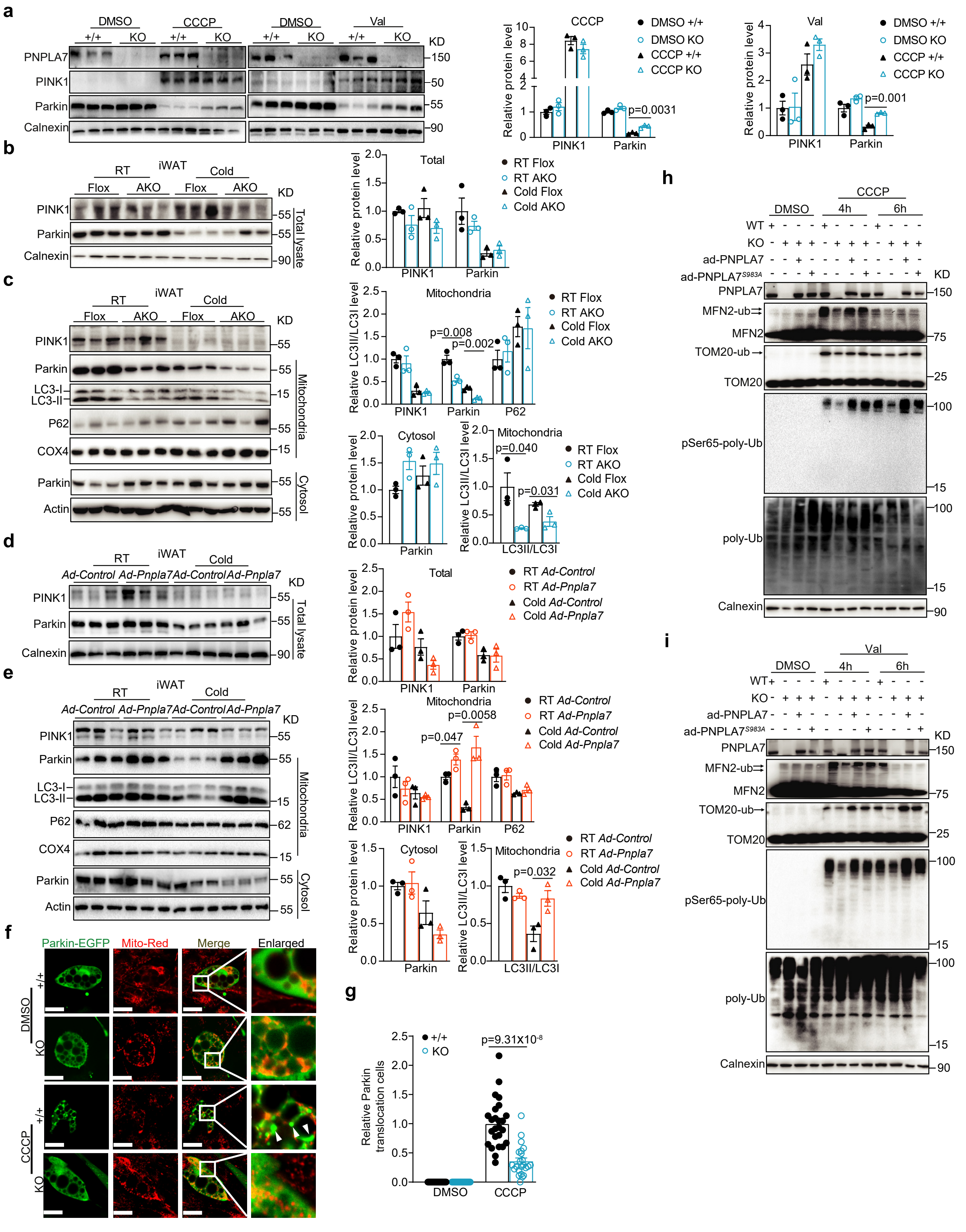
a
DMSO
CCCP
Val
+/+
KO
KD
PNPLA7
PINK1
Parkin
Calnexin
150
50
55
90
Relative protein level
DMSO +/+
DMSO KO
CCCP +/+
CCCP KO
p=0.0031
p=0.001
b
iWAT
RT
Cold
Flox
AKO
Total lysate
Total
RT Flox
RT AKO
Cold Flox
Cold AKO
c
LC3-I
LC3-II
P62
COX4
Actin
Mitochondria
Cytosol
Relative LC3II/LC3I level
p=0.008
p=0.002
p=0.040
p=0.031
LC3II/LC3I
d
Ad-Control
Ad-Pnpla7
RT Ad-Control
RT Ad-Pnpla7
Cold Ad-Control
Cold Ad-Pnpla7
e
p=0.0058
p=0.047
p=0.032
f
Parkin-EGFP
Mito-Red
Merge
Enlarged
g
Relative Parkin translocation cells
p=9.31X10-8
h
WT
ad-PNPLA7
ad-PNPLA7S983A
4h
6h
MFN2-ub
MFN2
TOM20-ub
TOM20
pSer65-poly-Ub
poly-Ub
i

Fig.S8. PNPLA7 deficiency reduces mitophagy by inhibiting Parkin mitochondrial translocation

(a) Representative Immunoblot results and densitometry analysis of PINK1, Parkin protein levels in differentiated mature adipocytes isolated from iWAT of 3-week-old control (+/+) and PNPLA7 knockout (KO) male mice (n=3 biological replicates). Data are presented as mean ± SEM. (Two-tailed Student's t test for 2-group comparisons). **(b, c)** Representative Immunoblot results and densitometry analysis of the indicated proteins in total lysate (**b**), mitochondrial fraction and cytosolic fraction (**c**) of iWAT harvested from control and $Pnpla7^{AKO}$ mice (n=3/group). Data are presented as mean ± SEM. (Two-tailed Student's t test for 2-group comparisons). **(d, e)** Representative Immunoblot results and densitometry analysis of the indicated proteins in total lysate (**d**), mitochondrial fraction and cytosolic fraction (**e**) of iWAT harvested from *Ad-control* and *Ad-Pnpla7* mice (n=3/group). Data are presented as mean ± SEM. (Two-tailed Student's t test for 2-group comparisons). **(f)** Colocalization of Parkin with mitochondria in differentiated adipocytes with or without PNPLA7 expression. Differentiated adipocytes from iWAT of control (+/+) and PNPLA7 knockout (KO) male mice were transfected with EGFP-Parkin for 48 h and cells were incubated with DMSO (vehicle) or CCCP (10 μM) for 3 h. Mitochondria were stained with Mito-tracker (red), EGFP-Parkin translocation to mitochondria was analyzed by confocal microscopy. (n=3

biological replicates). Scale bar=20 μm. **(g)** Quantification of the mitochondrial EGFP-Parkin positive cells as shown in (**f**). Colocalization of EGFP-Parkin with mitochondria was analyzed by Image J. (DMSO: n=24/group; CCCP: +/+ group n=25; KO group n=23). Data are presented as mean ± SEM. (Two-tailed Student's t test for 2-group comparisons). **(h, i)** Representative Immunoblot results of the indicated proteins in *Pnpla7* deficient mature adipocytes isolated from *Pnpla7* knockout male mice with or without infection with ad-*Pnpla7* or ad-$Pnpla7^{S983A}$ subjected to CCCP (10 μM) (**h**) and Valinomycin (1 μM) (**i**) treatment for the indicated time. (n=3 biological replicates).

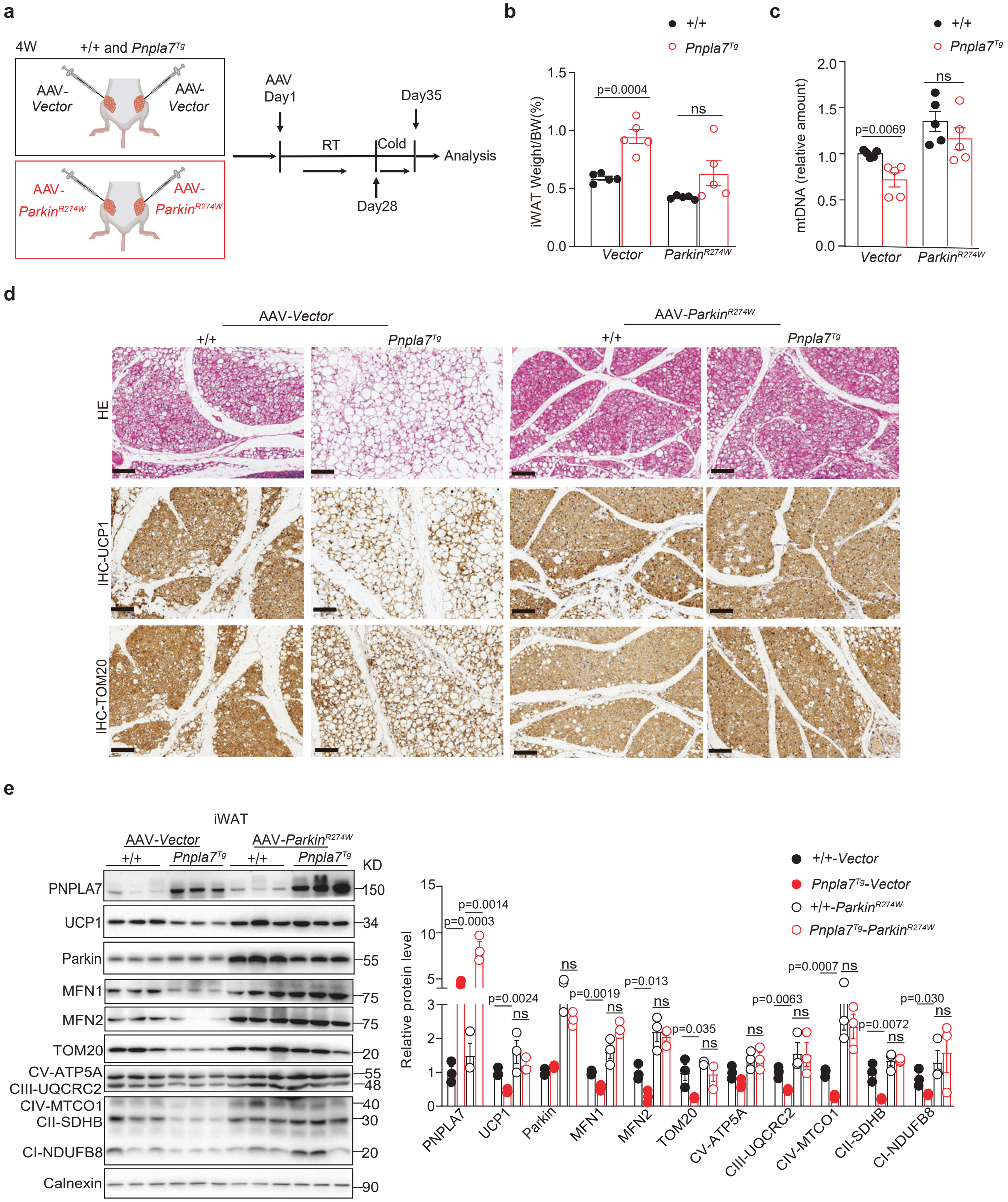
a
4W
+/+ and Pnpla7Tg
AAV-Vector
AAV-ParkinR274W
AAV Day1
Day35
RT
Cold
Analysis
Day28
b
+/+
Pnpla7Tg
iWAT Weight/BW(%)
p=0.0004
ns
Vector
ParkinR274W
c
mtDNA (relative amount)
p=0.0069
ns
d
AAV-Vector
AAV-ParkinR274W
HE
IHC-UCP1
IHC-TOM20
e
iWAT
KD
PNPLA7
UCP1
Parkin
MFN1
MFN2
TOM20
CV-ATP5A
CIII-UQCRC2
CIV-MTCO1
CII-SDHB
CI-NDUFB8
Calnexin
+/+-Vector
Pnpla7Tg-Vector
+/+-ParkinR274W
Pnpla7Tg-ParkinR274W
Relative protein level
p=0.0014
p=0.0003
p=0.0024
p=0.0019
p=0.013
p=0.035
p=0.0063
p=0.0007
p=0.0072
p=0.030

Fig.S9. PNPLA7 inhibits browning of iWAT by inhibiting Parkin mitochondrial translocation

(a) Illustration of *Parkin*^{R274W} overexpression in the inguinal WAT of *Pnpla7*^{Tg} *mice.* This illustration was created with Adobe Illustrator and BioRender.com, the mouse picture was created with BioRender.com. **(b)** Weight ratio of iWAT harvested from control (+/+) and *Pnpla7*^{Tg} (Tg) mice with or without overexpression *Parkin*^{R274W}. 4-week-old control (+/+) and *Pnpla7*^{Tg} (Tg) mice were injected with recombinant adeno-associated virus of control (Vector) or *Parkin*^{R274W} into the subcutaneous inguinal white adipose tissue and fed with normal chow diet for one month. Subsequently, these mice were exposed to 6 °C for 7 days and sacrificed. (n=5/group). Data are presented as mean ± SEM. (Two-tailed Student's t test for 2-group comparisons). **(c)** Relative mtDNA content of iWAT harvested from control (+/+) and *Pnpla7*^{Tg} (Tg) mice injected with AAV-*Vector* or AAV-*Parkin*^{R274W} described in (**a**). (n=5/group). Data are presented as mean ± SEM. (Two-tailed Student's t test for 2-group comparisons). **(d)** Representative H&E, UCP1 and TOM20 immunohistochemical staining images of iWAT sections from control (+/+) and *Pnpla7*^{Tg} (Tg) mice injected with AAV-vector (Vector) or AAV-*Parkin*^{R274W} described in (**a**). (n=3/group). Scale bar=100 μm. **(e)** Representative Immunoblot results and densitometry analysis of the indicated proteins in iWAT harvested from

Pnpla7^{Tg} mice injected with AAV-*Vector* or AAV-*Parkin*^{R274W}, respectively. (n=3/group). Data are presented as mean ± SEM. (Two-tailed Student's t test for 2-group comparisons).

Panel (a) Created in BioRender. Ji, X. (2025) https://BioRender.com/v1sk48t

Supplementary Table 1. qPCR Primers

Genes	Forward (5′→3′)	Reverse (5′→3′)
36B4	CACTGGTCTAGGACCCGAGAAG	GGTGCCTCTGGAGATTTTCG
Pnpla7	GAGCAACCACAGTGCATGCT	TGGGTACCTGCGCTTGATAGA
Ucp1	GAGGTGTGGCAGTGTTCATTG	GGCTTGCATTCTGACCTTCA
Pgc1-α	GGAGCTCCAAGACTCTAGACA	CCAAAGTCTCTCTCAGGTAGC
Prdm16	CAGCACGGTGAAGCCATTC	GCGTGCATCCGCTTGTG
CideA	CCGAGTACTGGGCGATACAGA	GGTTACATGAACCAGCCTTTGG
Dio2	CAGTGTGGTGCACGTCTCCAATC	TGACCAAAGTTGACCACCAG
Elovl3	TTCTCACGCGGGTTAAAAATGG	GAGCAACAGATAGACGACCAC
Ndufs3	TCCCAACTCGGCAGAAC	CCCGCTTTACCTCATCG
Sdhb	AGTTCTCATGCAGGCCTATC	CCAGCTGTTTGACACCAGAG
Uqcrc2	AAAGTTGCCCCGAAGGTTAAA	GAGCATAGTTTTCCAGAGAAGCA
Cox4	AGTGTTGTGAAGAGTGAAGAC	GCGGTACAACTGAACTTTCTC
Atp5b	GACTGGGATAAAGGTTGTGG	CCTGGGTAAAGCGGAAG
Tfam	GCAAAGGATGATTCGGCTCAGGGAA	CCGGATCGTTTCACACTTCGACGG
Tfb2m	GTTCTTTGGCAAGTGGCCTG	ACTGATTCCCCGTGCTTTGA
Nrf1	GGTGTTTGGCGCAGCACCTT	CTCTGGGATAAATG CCGAAGCT
Atgl	GAGAGAACGTCATCATATCCCACTT	CCACAGTACACCGGGATAAATGT
Hsl	GGAGCACTACAAACGCAACGA	TCGGCCACCGGTAAAGAG
Cpt1β	TTGCCCTACAGCTGGCTCATTTCC	GCACCCAGATGATTGGGATACTGT
Pparα	ACAAGGCCTCAGGGTACCA	GCCGAAAGAAGCCCTTACAG
Mcad	GCAACTGCCCGCAAGTTT	TACTCCCCGCTTTTGTCATATTC
Lcad	TCAATGGAAGCAAGGTGTTCA	GCCACGACGATCACGAGAT
Cox8b	TGTGGGGATCTCAGCCATAGT	AGTGGGCTAAGACCCATCCTG
Cox2	CAGTCCCCTCCCTAGGACTT	TTTCAGAGCATTGGCCATAGAA
Fasn	GCTGCGGAAACTTCAGGAAAT	AGAGACGTGTCACTCCTGGACTT

Supplementary Table 2. Antibodies information

Antibody	Host	Company	Catalog#	Dilution
PGC-1α	Rabbit	Abcam	ab3242	1:1000
UCP1	Rabbit	Fitzgerald	70R-UR001	1:2000
Calnexin	Rabbit	ENZO life	ADI-SPA-860	1:3000
MFN1	Mouse	Abcam	ab57602	1:1000
MFN2	Rabbit	Cell Signaling	9482S	1:1000
Tom20	Rabbit	Protientech	11802-1-AP	1:1000
Tim23	Mouse	Santa Cruze	sc-514463	1:1000
PINK1	Rabbit	Cell Signaling	6946S	1:1000
Parkin	Mouse	Cell Signaling	4211	1:1000
OXPHOS	Mouse	Abcam	ab110413	1:1000
LC3A	Rabbit	Cell Signaling	4599	1:1000
phospho-PKA substrate	Rabbit	Cell Signaling	96221S	1:1000
LC3B	Rabbit	Cell Signaling	2775	1:1000
Actin	Rabbit	Santa Cruze	sc-47778	1:1000
FACL-4	Rabbit	Santa Cruze	sc-365230	1:1000
COX4	Rabbit	Cell Signaling	11967S	1:1000
Tubulin	Mouse	Santa Cruze	sc-9104	1:1000
Lamin B	Mouse	Santa Cruze	sc-56144	1:1000
phospho-Ser65-ubiquitin	Rabbit	Merck Millipore	ABS1513-I	1:1000
Ubiquitin	Rabbit	Cell Signaling	43124	1:1000
HA	Rabbit	Cell Signaling	3724	1:1000
HA	Mouse	Sigma-Aldrich	9658	1:1000
Flag	Rabbit	Cell Signaling	F1804	1:1000
Flag	Mouse	Sigma-Aldrich	2368	1:1000
PNPLA7	Rabbit	Wang et al，2020	N/A	1:500
IgG	Rabbit	Cell Signaling	3900	1:1
IgG	Mouse	Cell Signaling	5415	1:1
IgG(H+L) Secondary Antibody, HRP	Rabbit	Jackson ImmunoResearch	115-035-003	1:10000
IgG(H+L) Secondary Antibody, HRP	Mouse	Jackson ImmunoResearch	111-005-003	1:10000
Alexa 568 Donkey	Goat	Molecular probes	A-11057	1:200
Alexa 568 Goat	Mouse	Molecular probes	A11031	1:200
