# Supplementary material for: PNPLA7 mediates Parkin-mitochondrial recruitment in adipose tissue for mitophagy and inhibits browning
Source: Nat Commun. 2025 Jul 19;16:6651. doi: 10.1038/s41467-025-61904-w (PMC12274619; doi:10.1038/s41467-025-61904-w)
Supplement: Supplementary file 2 — Reporting Summary [file 41467_2025_61904_MOESM2_ESM.pdf]

nature portfolio

Corresponding author(s): John Zhong Li

Last updated by author(s): May 15, 2025

Reporting Summary

Nature Portfolio wishes to improve the reproducibility of the work that we publish. This form provides structure for consistency and transparency in reporting. For further information on Nature Portfolio policies, see our Editorial Policies and the Editorial Policy Checklist.

Statistics

For all statistical analyses, confirm that the following items are present in the figure legend, table legend, main text, or Methods section.

n/a	Confirmed	
☐	☒	The exact sample size (n) for each experimental group/condition, given as a discrete number and unit of measurement
☐	☒	A statement on whether measurements were taken from distinct samples or whether the same sample was measured repeatedly
☐	☒	The statistical test(s) used AND whether they are one- or two-sided<br>*Only common tests should be described solely by name; describe more complex techniques in the Methods section.*
☐	☒	A description of all covariates tested
☐	☒	A description of any assumptions or corrections, such as tests of normality and adjustment for multiple comparisons
☐	☒	A full description of the statistical parameters including central tendency (e.g. means) or other basic estimates (e.g. regression coefficient) AND variation (e.g. standard deviation) or associated estimates of uncertainty (e.g. confidence intervals)
☐	☒	For null hypothesis testing, the test statistic (e.g. F, t, r) with confidence intervals, effect sizes, degrees of freedom and P value noted<br>*Give P values as exact values whenever suitable.*
☒	☐	For Bayesian analysis, information on the choice of priors and Markov chain Monte Carlo settings
☒	☐	For hierarchical and complex designs, identification of the appropriate level for tests and full reporting of outcomes
☒	☐	Estimates of effect sizes (e.g. Cohen's d, Pearson's r), indicating how they were calculated

Our web collection on statistics for biologists contains articles on many of the points above.

Software and code

Policy information about availability of computer code

Data collection	Olympus FV1200 confocal microscope, Comprehensive Lab Animal Monitoring System (CLAMS; TSE Phenomaster) , CalR2, Pannoramic MIDI (3DHISTECH) ,JEM-1010 electron microscope,QTRAP 6500 Plus spectrometers (SCIEX, Chromos, Singapore), XFe24(Seahorse Bioscience), ChemiDoc XRS+ system (Bio-Rad).
Data analysis	GraphPad Prism 9 , Image Lab (version 4.1) ,NIH ImageJ ,CaseViewer2.4 ,FV10-ASW software (4.0).

For manuscripts utilizing custom algorithms or software that are central to the research but not yet described in published literature, software must be made available to editors and reviewers. We strongly encourage code deposition in a community repository (e.g. GitHub). See the Nature Portfolio guidelines for submitting code & software for further information.

Data

Policy information about availability of data

All manuscripts must include a data availability statement. This statement should provide the following information, where applicable:

- Accession codes, unique identifiers, or web links for publicly available datasets
- A description of any restrictions on data availability
- For clinical datasets or third party data, please ensure that the statement adheres to our policy

All data generated or analyzed during this study are included in this article and its supplementary information files .Source data are provided as Source Data file and may be obtained from the corresponding authors upon request.

Research involving human participants, their data, or biological material

Policy information about studies with human participants or human data. See also policy information about sex, gender (identity/presentation), and sexual orientation and race, ethnicity and racism.

Reporting on sex and gender	N/A
Reporting on race, ethnicity, or other socially relevant groupings	N/A
Population characteristics	N/A
Recruitment	N/A
Ethics oversight	N/A

Note that full information on the approval of the study protocol must also be provided in the manuscript.

Field-specific reporting

Please select the one below that is the best fit for your research. If you are not sure, read the appropriate sections before making your selection.

☒ Life sciences ☐ Behavioural & social sciences ☐ Ecological, evolutionary & environmental sciences

For a reference copy of the document with all sections, see nature.com/documents/nr-reporting-summary-flat.pdf

Life sciences study design

All studies must disclose on these points even when the disclosure is negative.

Sample size	No statistical method was performed to predetermine sample size. Instead, sample sizes were determined based on reproducibility between biological replicates and individual experiments and on magnitude and consistency of measurable differences between groups. For in vivo experiments, cohort size was determined by types of experiment and availability of animals as littermates were used in each experiment and our sample size ranging from 4-10 mice per group are standard cohort size in this field.
Data exclusions	No data were excluded
Replication	All experiments were repeated biologically at least three times with similar results, as indicated in the figure legends.
Randomization	All animals were randomized into groups at the start of the study. For cell culture experiments, individual wells were randomized into groups of treatment conditions.
Blinding	Investigators were not blinded during experiments and outcome assessment. There were defined groups and the treatment to the exprimentsl need to be known to the person handling the mice/cells.

Reporting for specific materials, systems and methods

We require information from authors about some types of materials, experimental systems and methods used in many studies. Here, indicate whether each material, system or method listed is relevant to your study. If you are not sure if a list item applies to your research, read the appropriate section before selecting a response.

Materials & experimental systems

n/a	Involved in the study	
☐	☒	Antibodies
☐	☒	Eukaryotic cell lines
☒	☐	Palaeontology and archaeology
☐	☒	Animals and other organisms
☒	☐	Clinical data
☒	☐	Dual use research of concern
☒	☐	Plants

Methods

n/a	Involved in the study	
☒	☐	ChIP-seq
☒	☐	Flow cytometry
☒	☐	MRI-based neuroimaging

Antibodies

Antibodies used

1, Rabbit PGC-1α antibody, Abcam, Cat#ab313559. (1:1000 dilution for Immunoblotting)
2, Rabbit UCP1 antibody, Fitzgerald, Cat#70R-UR001. (1:2000 dilution for Immunoblotting and 1:200 for immunohistochemistry)
3, Rabbit Calnexin antibody, ENZO life, Cat# ADI-SPA-860. (1:2000 dilution for Immunoblotting)
4, Mouse MFN1 antibody, Abcam, Cat# ab126575. (1:1000 dilution for Immunoblotting)
5, Rabbit MFN2 antibody, Cell Signaling, Cat#9482S. (1:1000 dilution for Immunoblotting)
6, Rabbit Tom20 antibody, Proteintech, Cat#11802-1-AP. (1:1000 dilution for Immunoblotting and 1:200 for immunohistochemistry)
7, Mouse Tim23 antibody, Santa Cruze, Cat# sc-514463. (1:500 dilution for Immunoblotting)
8, Rabbit PINK1 antibody, Cell Signaling, Cat#6946S. (1:1000 dilution for Immunoblotting)
9, Mouse Parkin antibody, Cell Signaling, Cat#4211S. (1:1000 dilution for Immunoblotting)
10, Mouse OXPHOS antibody, Abcam, Cat# ab110413. (1:1000 dilution for Immunoblotting)
11, Rabbit LC3A antibody, Cell Signaling, Cat#4599S. (1:1000 dilution for Immunoblotting)
12, Rabbit phospho-PKA substrate antibody, Cell Signaling, Cat#9621S. (1:1000 dilution for Immunoblotting)
13, Rabbit LC3B antibody, Cell Signaling, Cat#2775S. (1:1000 dilution for Immunoblotting)
14, Mouse Actin antibody, Santa Cruze, Cat# sc-47778. (1:500 dilution for Immunoblotting)
15, Mouse FACL-4 antibody, Santa Cruze, Cat# sc-365230. (1:500 dilution for Immunoblotting)
16, Rabbit COX4 antibody, Cell Signaling, Cat#11967S. (1:1000 dilution for Immunoblotting)
17, Mouse Tubulin antibody, Santa Cruze, Cat# sc-9104. (1:1000 dilution for Immunoblotting)
18, Mouse Lamin B antibody, Santa Cruze, Cat# sc-56144. (1:1000 dilution for Immunoblotting)
19, Rabbit phospho-Ser65-ubiquitin antibody, Merck Millipore, Cat# ABS1513-I. (1:1000 dilution for Immunoblotting)
20, Rabbit Ubiquitin antibody, Cell Signaling, Cat#43124S. (1:1000 dilution for Immunoblotting)
21, Rabbit HA antibody, Cell Signaling, Cat#3724S. (1:1000 dilution for Immunoblotting)
22, Mouse HA antibody, Sigma-Aldrich, Cat# H9658. (1:1000 dilution for Immunoblotting)
23, Rabbit Flag antibody, Cell Signaling, Cat# 2368S. (1:1000 dilution for Immunoblotting)
24, Mouse Flag antibody, Sigma-Aldrich, Cat# F1804. (1:1000 dilution for Immunoblotting)
25, Rabbit PNPLA7 antibody, Wang et al 2020, Cat#N/A (1:500 dilution for Immunoblotting)
26, Rabbit IgG antibody, Cell Signaling, Cat#3900S. (1:1 dilution for immunoprecipitation)
27, Mouse IgG antibody, Cell Signaling, Cat#5415S. (1:1 dilution for immunoprecipitation)
28, Rabbit IgG(H+L) Secondary Antibody, HRP, Jackson ImmunoResearch, Cat#115-035-003. (1:10000 dilution for immunoprecipitation)
29, Mouse IgG(H+L) Secondary Antibody, HRP, Jackson ImmunoResearch, Cat#111-005-003. (1:10000 dilution for immunoprecipitation)
30, Goat Alexa Fluor 568 Donkey antibody, Invitrogen, Cat# A-11057. (1:200 dilution for Immunofluorescent)
31, Mouse Alexa Fluor 568 Goat antibody, Invitrogen, Cat# A11031. (1:200 dilution for Immunofluorescent)
31, Mouse Alexa Fluor 488 Donkey antibody, Invitrogen, Cat# A-21202. (1:200 dilution for Immunofluorescent)

Validation

https://www.abcam.cn/products/primary-antibodies/pgc1-alpha-antibody-epr25162-281-ab313559.pdf
https://www.biosynth.com/Files/Gen/SoftSpecification/70R-UR001
https://www.enzo.com/product/calnexin-polyclonal-antibody/pdf
https://www.abcam.cn/products/primary-antibodies/mitofusin-1-antibody-11e91h12-ab126575.pdf
https://media.cellsignal.com/coa/9482/4/9482-lot-4-coa.pdf
https://www.ptgcn.com/products/pictures/pdf/11802-1-AP.pdf
https://datasheets.scbt.com/sc-514463.pdf
https://media.cellsignal.com/coa/6946/7/6946-lot-7-coa.pdf
https://media.cellsignal.com/coa/4211/10/4211-lot-10-coa.pdf
https://www.abcam.cn/products/panels/total-oxphos-rodent-wb-antibody-cocktail-ab110413.pdf
https://www.cellsignal.com/products/primary-antibodies/lc3a-d50g8-xp-rabbit-mab/4599
https://media.cellsignal.com/coa/9621/16/9621-lot-16-coa.pdf
https://media.cellsignal.com/coa/2775/14/2775-lot-14-coa.pdf
https://datasheets.scbt.com/sc-47778.pdf
https://datasheets.scbt.com/sc-365230.pdf
https://media.cellsignal.com/coa/11967/11967-coa.pdf
https://www.scbt.com/zh/p/beta-tubulin-antibody-h-235
https://datasheets.scbt.com/sc-56144.pdf
https://www.merckmillipore.com/CN/zh/product/Anti-phospho-Ubiquitin-Ser65,MM_NF-ABS1513-I?ReferrerURL=https%3A%2F%2Fcn.bing.com%2F&bd=1#anchor_COA
https://media.cellsignal.com/coa/43124/4/43124-lot-4-coa.pdf
https://media.cellsignal.com/coa/3724/13/3724-lot-13-coa.pdf
https://www.sigmaaldrich.cn/specification-sheets/928/702/H9658-BULK_________SIGMA_____.pdf
https://media.cellsignal.com/coa/2368/12/2368-lot-12-coa.pdf
https://www.sigmaaldrich.cn/specification-sheets/469/360/F1804-5MG__________SIGMA_____.pdf
https://media.cellsignal.com/coa/3900/53/3900-lot-53-coa.pdf
https://media.cellsignal.com/coa/5415/14/5415-lot-14-coa.pdf
https://www.jacksonimmuno.com/catalog/products/115-035-003
https://www.jacksonimmuno.com/catalog/products/111-005-003
https://assets.thermofisher.cn/TFS-Assets%2FBID%2Fcertificate%2FCertificates-of-Analysis%2FA11057%20Lot%202563881%20CofA.pdf
https://assets.thermofisher.cn/TFS-Assets%2FBID%2Fcertificate%2FCertificates-of-Analysis%2FA11031%20Lot%202424253%20CofA.pdf
https://assets.thermofisher.cn/TFS-Assets%2FBID%2Fcertificate%2FCertificates-of-Analysis%2FA21202%20Lot%202018296%

20CofA.pdf

Eukaryotic cell lines

Policy information about cell lines and Sex and Gender in Research

Cell line source(s)	HEK293T, HEK293A and 3T3-L1 cells were gift from Prof. Peng Li (Zhengzhou University). All primary adipocytes were isolated form inguinal WAT of 3-4 week old male mice
Authentication	We did not authenticate in-house. Cells were gift from Prof. Peng Li (Zhengzhou University) and propagated for use in our lab.
Mycoplasma contamination	All cell lines were tested negative for mycoplasma contamination.
Commonly misidentified lines (See ICLAC register)	No cell lines used in this study were found in the database of commonly misidentified cell lines maintained by ICLAC and NCBI

Animals and other research organisms

Policy information about studies involving animals; ARRIVE guidelines recommended for reporting animal research, and Sex and Gender in Research

Laboratory animals	1. Mouse, PNPLA7-Tg(Ap2-promoter)<br>2. Mouse, PNPLA7-AKO(Adiponectin-Cre)<br>3. Mouse, PNPLA7-KO(CRISPR-Cas9 system)<br>4.Mouse, Adiponectin-Cre<br>5.Mouse, C57BL/6J<br>8-week-old male mice were used for experiments where 8-week-old male mice were fed a normal chow diet (NCD) under room temperature ?25??or cold temperature(6?) for 1 weeks. Mice were fed a NCD and were kept on a 12-h light/12-h dark cycle with free access to food and water i.
Wild animals	No wild animals were used in this study.
Reporting on sex	Although the mice used in this study were male mice. Our research findings are not limited to a specific gender, as sex was not a factor in our study design involving vertebrates, cell lines. We have no data that should be reported disaggregated for sex
Field-collected samples	No field-collected samples were used
Ethics oversight	All animal protocols were reviewed and approved by the Animal Ethics Committee of the Nanjing Medical University and followed the National Institute of Health guidelines on the care and use of animals.

Note that full information on the approval of the study protocol must also be provided in the manuscript.

Plants

Seed stocks	N/A
Novel plant genotypes	N/A
Authentication	N/A
